# Design principles for shift current photovoltaics

**DOI:** 10.1038/ncomms14176

**Published:** 2017-01-25

**Authors:** Ashley M. Cook, Benjamin M. Fregoso, Fernando de Juan, Sinisa Coh, Joel E. Moore

**Affiliations:** 1 Department of Physics, University of California, Berkeley, California 94720, USA; 2 Department of Physics, University of Toronto, Ontario, Canada, M5S 1A7; 3 Materials Sciences Division, Lawrence Berkeley National Laboratory, Berkeley, California 94720, USA

## Abstract

While the basic principles of conventional solar cells are well understood, little attention has gone towards maximizing the efficiency of photovoltaic devices based on shift currents. By analysing effective models, here we outline simple design principles for the optimization of shift currents for frequencies near the band gap. Our method allows us to express the band edge shift current in terms of a few model parameters and to show it depends explicitly on wavefunctions in addition to standard band structure. We use our approach to identify two classes of shift current photovoltaics, ferroelectric polymer films and single-layer orthorhombic monochalcogenides such as GeS, which display the largest band edge responsivities reported so far. Moreover, exploring the parameter space of the tight-binding models that describe them we find photoresponsivities that can exceed 100 mA W^−1^. Our results illustrate the great potential of shift current photovoltaics to compete with conventional solar cells.

## Abstract (web summary)

Above band-gap photovoltage could be achieved in materials with a net polarization. Here, Cook *et al*. compute the contribution to the shift current from the band edge and identify two classes of shift current photovoltaics materials, GeS and ferroelectric polymer films.

Cost-effective, high-performing solar cell technology is an essential piece of a sustainable energy strategy. Exploring approaches to photo-current generation beyond conventional solar cells based on pn junctions is worthwhile given that their performance is in practice constrained by the Shockley–Queisser limit^1^. One of the most promising alternative sources of photocurrent is the bulk photovoltaic effect (BPVE) or ‘shift current' effect, a nonlinear optical response that yields net photocurrent in materials with net polarization^2^,^3^,^4^,^5^,^6^,^7^,^8^,^9^,^10^,^11^. Contrary to conventional pn junctions, the BPVE is able to generate an above band-gap photovoltage^12^, potentially allowing the performance of BPVE-based photovoltaics to surpass conventional ones. However, closed-circuit currents generated via the BPVE reported in the literature have typically been small compared with those generated in pn junction photovoltaics^13^,^14^,^15^. Recent interest in the BPVE also stems from the proposal that it may be at work in a promising class of materials for photovoltaics known as hybrid perovskites^13^, an extremely active field of research^16^,^17^,^18^,^19^,^20^,^21^,^22^,^23^,^24^,^25^,^26^,^27^,^28^,^29^.

The fundamental requirement for a material to produce a current via the BPVE is that it breaks inversion symmetry, allowing an asymmetric photoexcitation of carriers. But despite considerable case-by-case study of the BPVE, the necessary ingredients to optimize a BPVE-based solar cell are not sufficiently well understood. As with conventional solar cells, band gaps in the visible (1.1–3.1 eV)^15^,^30^ and large electronic densities of states^14^,^31^ are always beneficial. In addition, to produce a solar cell that responds to unpolarized sunlight, a highly anisotropic material must be used, since otherwise there is no preferred direction for the current to flow. But beyond these natural requirements, our only guiding knowledge is that the shift current depends explicitly on the nature of the electronic wavefunctions^31^,^32^ and that it is not correlated with the material polarization in any obvious way^15^ despite the fact that both shift currents and polarization originate from inversion symmetry breaking.

In the current situation, a more generic understanding of what makes the BPVE strong is highly desirable. When tackling complex material science problems, stripping off all complications and optimizing the simplest model that captures the relevant physics often proves the best strategy, as shown for example in thermoelectricity studies^33^,^34^,^35^. In this work, we present simple design principles for BPVE optimization based on the study of an effective model for the band edges. With this model, the band edge shift current is given by the product of the joint density of states (JDOS) and a matrix element, both given by simple expressions in terms of a few model parameters. The simplicity of the model allows us to derive the main principle that band edges with semi-Dirac type of Hamiltonians are the best starting point to obtain large band edge prefactors. In addition, by relating the effective model parameters to realistic tight-binding models, we can predict that several materials with the required band structure have larger shift currents than any reported so far.

## Results

### Density of states in one- and two-dimensions

**Figure 1:**
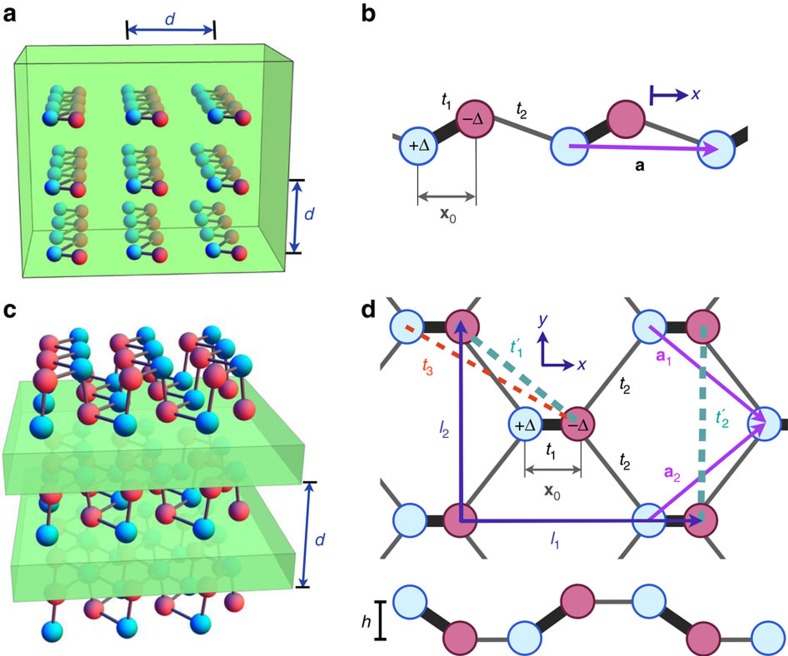
Schematics of proposed shift current photovoltaics: (**a**) Three-dimensional (3D) structure of a solar cell built by stacking one-dimensional ferroelectric polymers. (**b**) Simplified two-band tight-binding model of a polymer. (**c**) 3D structure of a solar cell made by stacking two-dimensional monolayers of a monochalcogenide. The inert spacers between layers prevent the restoration of bulk inversion symmetry. (**d**) Simplified two-band tight-binding model for a monochalcogenide layer.

In our search for materials we should look for large JDOS in systems where the band edge is closely aligned with the peak of the solar spectrum, around 1.5 eV. Since the band edge always induces a Van Hove singularity in the density of states, the requirement of a large peak in the photoresponse can be naturally better satisfied by low-dimensional materials, which generically present stronger singularities^36^. Materials of one and two dimensions are therefore the focus of this work. Among one-dimensional materials, ferroelectric polymers are suitable candidates for shift-current photovoltaics: they strongly break inversion symmetry, some have suitable band gaps for photovoltaics applications^37^,^38^,^39^,^40^, and they can be produced in macroscopically oriented samples. For these reasons, we consider solar cells consisting of such polymer films, shown in Fig. 1a. Two-dimensional materials^41^ also have great potential for photovoltaics, as shown by demonstration of a pn-junction photovoltaic effect in dichalcogenide heterostructures^42^,^43^,^44^, and in few-layer black phosphorus^45^. However, these well known two-dimensional (2D) semiconductors have vanishing shift currents because of either inversion or rotation symmetry. Group IV monochalcogenides have emerged in the past years as a new familiy of inversion-breaking, anisotropic 2D materials with fascinating properties^46^,^47^,^48^,^49^,^50^, and interest in growing as thin films of all four members of the family, GeS^51^,^52^,^53^,^54^, GeSe^53^,^54^, SnS^55^,^56^ and SnSe^57^,^58^,^59^ has now been isolated experimentally. In this work, we show that GeS is ideally suited to realize high values of the BPVE. Their GeS structure is shown in Fig. 1c.

To understand how to optimize the photoresponse, we first discuss how the shift current can be computed for a tight-binding model, and then we proceed to apply this formalism to describe a generic band edge and the response of particular materials.

### Shift current

In this work we consider the shift current contribution to the BPVE and we shall use both terms interchangeably (note the BPVE can have other contributions as well^6^). With electric field *E*_*b*_(*ω*) at frequency *ω* and linearly polarized in the *b* direction, the shift current is a DC response of the form^6^


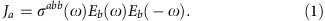


Defining an intensity for each polarization, 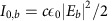, we define the photoresponsivity *κ*^*abb*^ as the current density generated per incident intensity *J*_*a*_=*κ*^*abb*^*I*_0,*b*_, which gives 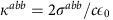. Note that in conventional solar cells the current is also linear with intensity. For a *D*-dimensional system, *κ*^*abb*^ takes the form^7^,^9^


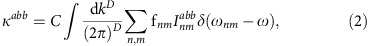


where 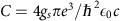, with *c* being the speed of light, 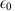 the vacuum permittivity and *g*_*s*_=2 accounts for the spin degeneracy. In what follows we set *ħ*=1. Summation of indices is explicitly indicated using the summation symbol. The sum is over all Bloch bands, with *ω*_*nm*_=*E*_*n*_−*E*_*m*_ the energy difference between bands *n* and *m* and f_*nm*_=*f*_*n*_−*f*_*m*_ the difference of Fermi occupations, which we take at zero temperature. The integrand is


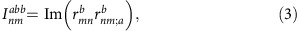


where 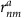 are the inter-band matrix elements of the position operator (or inter-band Berry connections), defined as 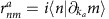 for *n*≠ *m* and zero otherwise, where 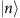 is the eigenstate of band *n*. A semicolon denotes a generalized derivative 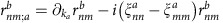, where 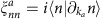 is the diagonal Berry connection for band *n*.

### Generic two-band model

With the aim of describing the shift current response of the band edge of a semiconductor, next we consider the shift current of a generic two-band model. The Fourier transform of the real space Hamiltonian is performed with the choice of phases 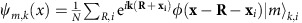, where *φ*(**x**) is a localized orbital and **x**_*i*_ is the position of site *i* in the unit cell. This choice is made in order to naturally incorporate the action of the position operator, see refs 60, 61, 62. The Hamiltonian matrix takes the form


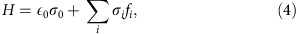


where *σ*_0_ is the identity matrix, *σ*_*i*_=*σ*_*x*_, *σ*_*y*_, *σ*_*z*_ are the Pauli matrices and 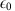 and *f*_*i*_=*f*_*x*_, *f*_*y*_, *f*_*z*_ are generic functions of momenta **k** (the momentum label is omitted to simplify notation). The conduction and valence bands are given by *E*_1_=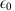
+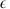, *E*_2_=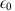
−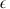, respectively and 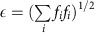. Note that this basis choice implies that the Hamiltonian matrix elements are not periodic in the Brillouin Zone, *H*_*ij*_(**k**+**G**)≠*H*_*ij*_(**k**) with **G** a reciprocal lattice vector.

To compute the shift current, the direct use of equation (3) requires the evaluation of derivatives of Bloch functions, which can be difficult to compute numerically. Previous works^4^,^7^,^9^ have addressed this problem with the use of identities that replace wavefunction derivatives with sums over all states of matrix elements of Hamiltonian derivatives. These identities are known as sum rules and rely on the fact that momentum and velocity operators are proportional in the plane wave basis *p*=*mv*, which is not true in the tight-binding formalism. In this work we derived a generalized sum rule appropriate for tight-binding models (see Methods section), from which the integrand equation (3) can be evaluated for any two-band model in terms of the Hamiltonian derivatives only. The result is


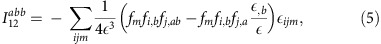


where the compact derivative notation 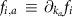 and 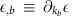 is used. Equation (5) is one of the main results of this work. Several general principles to maximize the band edge shift current can be derived from this expression. A straightforward one is that, since this expression does not depend on 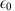, particle–hole asymetry does not influence the shift current at all. Therefore 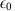 is set to zero from now on. The additional term that appears only for tight-binding models in this more general sum rule is *f*_*m*_ *f*_*i*,*b*_ *f*_*j*,*ab*_, which is absent in previous formulations. For a direct band gap, this term dominates the response exactly at the band edge, since to lowest order in *k* the first term always has constant contribution, while the second one is at least linear in *k* for any model due to the energy derivative 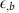. For this term to be finite, the three Pauli matrices in the Hamiltonian must have constant, linear and quadratic coefficients, in any order. Satisfying this low-energy constraint can be taken as another general principle in the search for materials with large shift current.

More explicit guidelines can be obtained by considering an explicit low-energy model with a direct band gap at a time reversal invariant momentum. Expanding the Hamiltonian around it we get


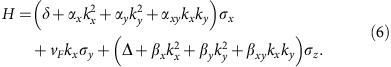


Time reversal symmetry H*(−**k**)=*H*(**k**) prevents quadratic terms in *σ*_*y*_, and we have taken the linear term to be in the *x* direction without loss of generality. Note this type of linear term requires the breaking of any *C*_*n*_ rotation symmetry with *n*>2. The band gap of this model is 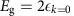. Evaluating equation (5) we get


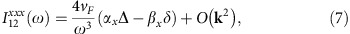



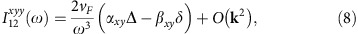


while 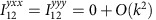. Also note that in order to have a non-zero shift current quadratic terms in *σ*_*x*_ or *σ*_*z*_ are required. In 2D, the fact that *I*^*xyy*^ is in general non-zero means that the current need not be in the direction of the electric field polarization.

The shift current close to the band edge can now be obtained by substituting equations (7) and (8) into equation (2), which gives


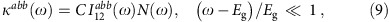


where 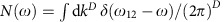 is the JDOS. Equation (9) provides an analytical formula for *ω* close to the band edge for a very general class of models. This simple expression allows one to disentangle the contributions of the shift current integrand and the JDOS and hence to optimize them independently.

To maximize the response we therefore require band structures where the JDOS has a strong singularity. It is well known that in the one-dimensional (1D) case, the generic JDOS diverges as a square root, *N*(*ω*)∝(*ω*−*E*_g_)^−1/2^. 1D systems such as polymers or nanowires or systems in the quasi-1D limit will in general have a large response. In 2D, the band edge JDOS has a finite jump of *N*(*ω*)=(*m*_*x*_*m*_*y*_)^1/2^/2*π*, where *m*_*i*_ are the average effective masses for valence and conduction bands. A singular *N*(*ω*) thus occurs in 2D when the inverse effective mass vanishes. In the effective model in Equation (6), this happens when *δ*=0, which realizes what we may call a gapped semi-Dirac dispersion^63^, since the coefficients of *σ*_*y*_ and *σ*_*x*_ are linear and quadratic in momentum, respectively. In such a case we have *N*(*ω*)∝(*ω*−*E*_g_)^−1/4^ (full expressions for *N*(*ω*) may be found in the Methods section).

For materials with large JDOS, the current can be further enhanced by appropriately tuning the parameters in Equations (7) and (8). This is most easily discussed if these parameters can be related to microscopic lattice models. In the next section, we discuss tight-binding models for simple materials that realize the described types of band structures.

### Material realizations and lattice models

As a realization of the 1D case, we consider ferroelectric polymers that break inversion symmetry such as polyvinylidene fluoride or disubstituted polyacetilene^39^,^40^,^64^. This system is described by the tight-binding model schematically shown in Fig. 1b, defined in terms of two types of hoppings, *t*_1_ and *t*_2_, alternating on-site potentials ±Δ, and orbital centres at *x*=0 and *x*=*x*_0_. With our choice of basis functions, the Hamiltonian is specified by 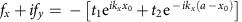 and *f*_*z*_=Δ, where *a*=10 Å is the lattice constant and the distance between closest neighbours is ref. 64 *x*_0_=0.48*a*. For estimates of the tight-binding parameters, we consider the example of disubstituted polyacetilene that was experimentally realized in ref. 39, with a band gap of 2.5 eV. For regular polyacetilene, where Δ=0, the hopping parameters and band gap have been estimated as ref. 64 *t*_1_=2.85 eV, *t*_2_=2.15 eV, *E*_g_=1.4 eV. Assuming the same hopping for the disubstituted version, we use Δ=1.0 eV to match the observed band gap. Note that the dispersion does not depend on *x*_0_.

Using Equations (2) and (5) we can now compute the shift current for this 1D model. Expanding about the low energy momentum *k*_*x*_=*π*/*a* and performing a constant rotation of the Pauli matrices, we obtain an effective model as equation (6) with parameters *k*_*y*_=0 and *δ*=*t*_1_−*t*_2_, *v*_*F*_=(*t*_1_−*t*_2_)*x*_0_+*t*_2_*a*, *α*_*x*_=[*t*_2_(*a*−*x*_0_)^2^−*t*_1_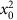]/2.

**Figure 2:**
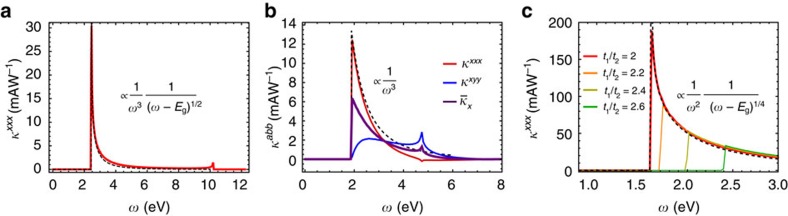
Frequency dependence of photoresponsivity. (**a**) Responsivity for a stack of disubstituted polyacetylene polymers with tight-binding parameters *t*_1_=2.85, *t*_2_=2.15, Δ=1.0 in eV, showing the square root divergence of the current at the band edges. (**b**) Various non-zero components of the responsivity tensor for a stack of 2D monochacogenides with parameters *t*_1_=−2.33, *t*_2_=0.61, *t*_3_=0.13, Δ=0.41 in eV and *x*_0_=0.52 Å. A large peak is observed in *κ*^*xxx*^ at the band edge. (**c**) Responsivity for Δ=0.8 eV, *x*_0_=0.6 Å, *t*_3_=0 and different hopping ratios |*t*_1_|/*t*_2_ approaching the semi-Dirac limit. The emergence of a singularity is observed. In the three figures, solid lines show the shift current components as computed from the tight-binding model, and a dashed line in each subfigure shows the *xxx* shift current component as predicted by the effective low energy model valid near the edge equation (9).

To be able to compare the responsivity of these materials to that of a three-dimensional system, we consider a stack of polymers as depicted in Fig. 1a, separated by a distance *d* which we take to be equal to the lattice constant of the polymer *d*=*a*. The photoresponsivity is then 
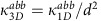
. The typical photoresponsivity spectrum of this model with this convention is shown in Fig. 2a.

For the 2D case, we require a layered material that breaks both inversion and rotational symmetries. The most popular of the recently isolated 2D semiconductors break either inversion (BN, MoS_2_) or rotational symmetries (black phosphorus^65^, Re*S*_2_ (ref. 66)), but not both. An inversion symmetry breaking version of the strongly anisotropic black phosphorus, a group V element, can be obtained combining elements of the IV and VI groups. These group IV monochalcogenides, such as GeS, are predicted to be stable in the monolayer form with the orthorhombic structure of black phosphorus^46^,^47^.

These materials can be described with a tight-binding model similar to the one used for black phosphorus^67^,^68^,^69^. While the GeS unit cell contains two Ge–S pairs at different heights, a unit cell with a single Ge–S pair can be used when the physics to be probed is insensitive to the heights of the atoms (see Methods for a detailed explanation). The two band Hamiltonian is specified by 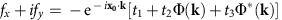, where **x**_0_=(*x*_0_, 0) and 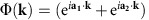, and *f*_*z*_=Δ. **a**_1_ and **a**_2_ are the lattice vectors. See Fig. 1d for the definition of the hopping integrals. Again note the dispersion is independent of *x*_0_. The specific values of the tight-binding parameters for GeS have been obtained by fitting an *ab-initio* calculation as described in the Methods section, where the coefficients of the low-energy model near the band edge are also shown. Note in this lattice structure there is a mirror symmetry *y*→−*y*, which is represented as the identity, and restricts *α*_*xy*_=*β*_*xy*_=0. (This is so because both conduction and valence bands are even under the symmetry, as it also happens in black phosphorus. This is also the result of our *ab-initio* calculation.) This symmetry still allows a linear term of the form *k*_*x*_*σ*_*y*_, crucial for the semi-Dirac type of band structure. In this model, the semi-Dirac limit is realized when *t*_1_=−2(*t*_2_+*t*_3_)^70^.

We consider a stack of monolayers separated by *d*=*a*, as shown in Fig. 1c. In this case, we consider an inert spacer layer between the GeS layers to avoid the restoration of inversion symmetry that would occur if we were to stack GeS into its natural bulk form. The three-dimensional photoresponsivity of this model, given by 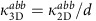, is computed using equations (2) and (5). To make contact with the 1D case we consider a stacking distance *d*=*a*≡(|**a**_1_|+|**a**_2_|)^1/2^ and *x*_0_=0.18*a*. The results are shown in Fig. 2b. We see that both *κ*^*xxx*^ and *κ*^*xyy*^ are in general finite, and the polarization average is also finite due to the strong anisotropy.

The response of the monochalcogenides is large because they are close in parameter space to the gapped semi-Dirac Hamiltonian. This is best illustrated by considering the evolution of a fictitious system where the hoppings are tuned (with *t*_3_=0 for simplicity) to the semi-Dirac case |*t*_1_|/*t*_2_=2, where the divergence of the response is clearly appreciated. This evolution is shown in Fig. 2c.

### Further optimization

**Figure 3:**
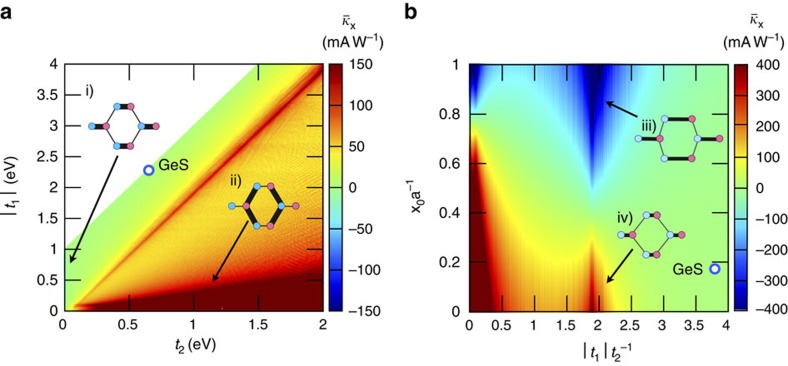
Phase diagrams for monochalcogenide layer tight-binding model. (**a**) Polarization-averaged photoresponsivity in the *x*-direction, 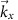, at the band gap frequency plotted as a function of hopping parameters |*t*_1_| and *t*_2_, keeping the band gap fixed at 1.89 eV by tuning Δ accordingly. The Ge-S distance is *x*_0_=0.52 Å and *t*_3_=0. The location of GeS on the phase diagram is marked by a white circle with blue outline. Regions for which the gap cannot be kept at 1.89 eV are left white. (i) and (ii) show bond strengths in the limits where 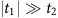 and 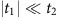, respectively, to illustrate the two extremes of the phase diagram. (**b**) Polarization-averaged photoresponsivity in the *x*-direction, 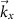, at the band gap frequency plotted as a function of the Ge-S distance *x*_0_ in units of 
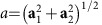
 and ratio of hopping parameters |*t*_1_|/*t*_2_. Here, Δ and *t*_2_ are set to GeS values of 1.1 and 0.61 eV, respectively. The location of GeS on the phase diagram is marked by a white circle with blue outline. (iii) and (iv) show two extreme cases of the phase diagram, where *x*_0_ is large and small, respectively.

After describing the representative tight-binding models with large JDOS, we may now address a more systematic analysis of the photoresponsivity. First, we consider exploring the phase diagram of the monochalcogenides by sweeping |*t*_1_|, *t*_2_ in parameter space while the band gap is fixed at 1.89 eV by choosing Δ appropriately and *t*_3_=0 for simplicity. Figure 3a shows the polarization averaged photoresponsivity, 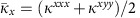, for the parameters *x*_0_=0.18*a* and *θ*=0.69. This phase diagram summarizes nicely the most physically relevant regimes where the shift current is large due to a divergent JDOS, namely the 1D limit where 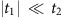, and the semi-Dirac regime where 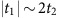. In this phase diagram, the point corresponding to *t*_1_ and *t*_2_ of GeS is shown as a white circle with blue outline.

Next we illustrate a very important feature of the behaviour of the shift current integrand. Equations (7) and (8) depend generically on the hoppings and lattice parameters. The energy does not depend on the parameter *x*_0_, but the wavefunctions do. In Fig. 3b, we show the peak photoresponsivity as a function of |*t*_1_|/*t*_2_ and *x*_0_. A large response is observed in the semi-Dirac limit 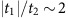. However, a very strong dependence on *x*_0_ and even a sign change is also observed. The dependence on *x*_0_ dramatically illustrates the fact that the shift current depends not only on the band structure but also on the wavefunctions. This can be seen explicitly in the fact that the effective mass 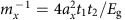 is independent of *x*_0_, but the combination *v*_*F*_*α*_*x*_ appearing in the shift current integrand is not. In particular *α*_*x*_ vanishes for 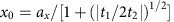, which means that regardless of the JDOS, the band edge response can actually be zero. This behaviour is characteristic of Berry connections, which depend explicitly on the positions of the sites in the unit cell.

## Discussion

In this work, we have shown how an effective model for the band edge enables a clean separation of the two factors that contribute to a large shift current: the standard JDOS and the shift current matrix element. This model also allows us to readily identify materials with semi-Dirac-like Hamiltonians as those where both factors can be made large. Several other general conclusions can be drawn from the form of the effective shift current integrand in equations (7) and (8). First, since the 1/*ω*^3^ factor becomes 1/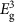 at the band edge, materials with smaller gaps are expected to have larger shift currents. A second conclusion is that while looking for materials with large JDOS is a good guiding principle, the shift current integrand depends on other microscopic details that can change the response dramatically. Within our simple model, the shift current can be maximized by bringing the two sites of the unit cell closer together, which is a requirement that the monochalcogenides satisfy well. Materials that may perform even better than GeS may be searched for exploring different chemical compositions, alloying or by strain engineering.

Our results were made possible by the derivation of a new sum rule appropriate for tight-binding models. With this sum rule, our work can be easily extended to tight-binding models with more than two bands, or systems where the minimum direct gap is not at a time-reversal invariant momentum. We expect that the formalism developed here will provide the necessary link to combine *ab-initio* methods with effective models, allowing for more in-depth, systematic study of shift current photovoltaics.

Our results should be compared with known ferroelectric materials that have been recently studied. In the visible range of frequencies, *ω*⩾3 eV, we find peak values of 0.1 mAW^−1^ in BiFeO_3_ (ref. 30), 1 mAW^−1^ in hybrid perovskites^13^ and a maximum 10 mAW^−1^ in BaTiO_3_ (ref. 14) or NaAsSe_2_ (ref. 15). The realistic materials that we propose present larger responsivities, with the additional advantage that the peak is by construction at the band edge. Moreover, as Figs 2c and 3b show, peak responses on the order of several hundreds of mAW^−1^ could be achieved with materials closer to the semi-Dirac regime. To compare with conventional photovoltaic mechanisms, the total current per intensity of a crystalline Si solar cell exposed to sunlight is about 400 mAW^−1^ (ref. 71).

Given these numbers, our work is a sign that shift current photovoltaics capable of surpassing conventional solar cells may be close at hand, and a push to investigate their full potential using methods discussed in this work—along with established techniques—is warranted. We believe that the simple principles derived in our work will serve as a guide for both theory and experiment in the development and optimization of the next generation of shift current photovoltaics.

## Methods

### Shift current

To make contact with previous work, we note the shift current integrand in equation (3) is sometimes expressed in terms of the phase of the inter-band matrix element 
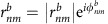
 as 
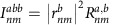
 where


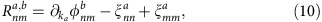


is known as the shift vector. The response to a natural light source such as sunlight, which is unpolarized, is obtained by averaging *κ*^*abb*^ over polarization. Taking 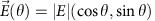 we have


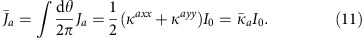


### Sum rule

The expression for the shift current presented in the main text can be obtained by the use of a sum rule for the quantity 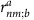, which is obtained from the identity


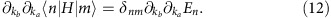


Evaluating both sides explicitly for *n*≠*m*, the identity can be expressed as


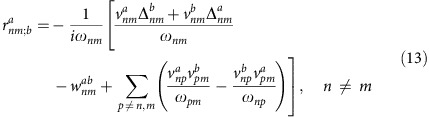


where 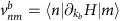 are the velocity matrix elements, 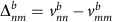, 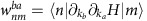 and *ω*_*nm*_=*E*_*n*_−*E*_*m*_. In the evaluation, we used


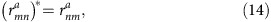



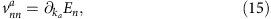



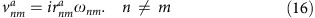


The first equality follows from 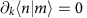 if *m*≠*n*, while the last two follow from 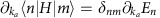. Note this sum rule contains the extra term 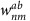 compared with ref. 9, where *H*=*p*^2^/2*m*+*V*(*x*) and 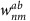
=*δ*_*nm*_*δ*^*ab*^/*m*, which has no off diagonal component. Quite importantly, the term 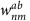 in tight-binding models is the one responsible for all band edge contributions. Also note that it has been argued before that *I*^*xxx*^=0 for a two-band model^4^, which is actually only true if 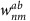
=0.

### Two-band model

For the case of two bands, *m*=1, *n*=2 the use of the sum rule for the shift current integrand in equation (3) leads to the simplified expression


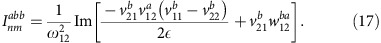


To evaluate this expression we compute the wave functions of *H*


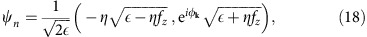


with *n*=1, 2, *η*=(−1)^*n*^, and *φ*_**k**_=arctan(*f*_*y*_/*f*_*x*_). The required matrix elements are


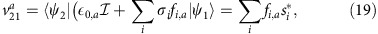



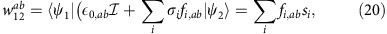


where the off diagonal matrix element 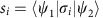 is


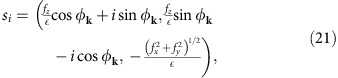


and the diagonal velocity matrix elements are computed from equation (15). The imaginary part in equation (17) can be taken using 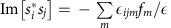 and this leads to equation (5) in the main text.

### Joint density of states

To compute the JDOS, we first start with the 1D case. Close to the band edge, we expand the energies of conduction and valence bands as *E*_*i*_≈*E*_*i*_(0)+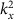/2*m*_*i*,*x*_, so that *ω*_12_=*E*_1_−*E*_2_≈*E*_g_+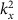/2*m*_*x*_ where the total effective mass 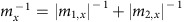 is given by


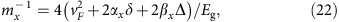


and solve for 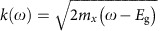. Rescaling 2*m*_*x*_ we get


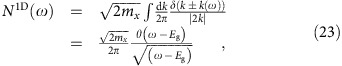


where we get the expected 1D singularity. For the generic 2D case, again we expand *ω*_12_≈*E*_g_+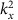/2*m*_*x*_+*k*_*y*_^2^/2*m*_*y*_, where *m*_*x*_ is still given by equation (22) and


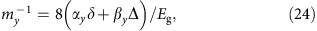


We consider the case when *m*_*x*_>0, *m*_*y*_>0, so that the minimum does lie at 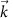
=0. By rescaling 2*m*_*x*_ and 2*m*_*y*_ we get in polar coordinates


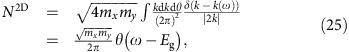


which is the expected constant result. Finally, the semi-Dirac case occurs in 2D when 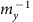
=0, which in the absence of second neighbour hopping occurs exactly at *δ*=0. In this case, we keep the complete expression for *ω*_12_=((*α*_*x*_
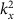
+*α*_*y*_*k*_*y*_^2^)^2^+*v*_*F*_^2^
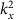
+Δ^2^)^1/2^. In polar coordinates we have


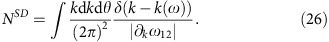


We now rescale *α*_*x*_, *α*_*y*_ instead, solve for *k*


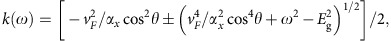


and get


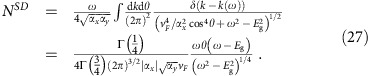


### *Ab-initio* calculation and tight-binding fit for GeS

**Figure 4:**
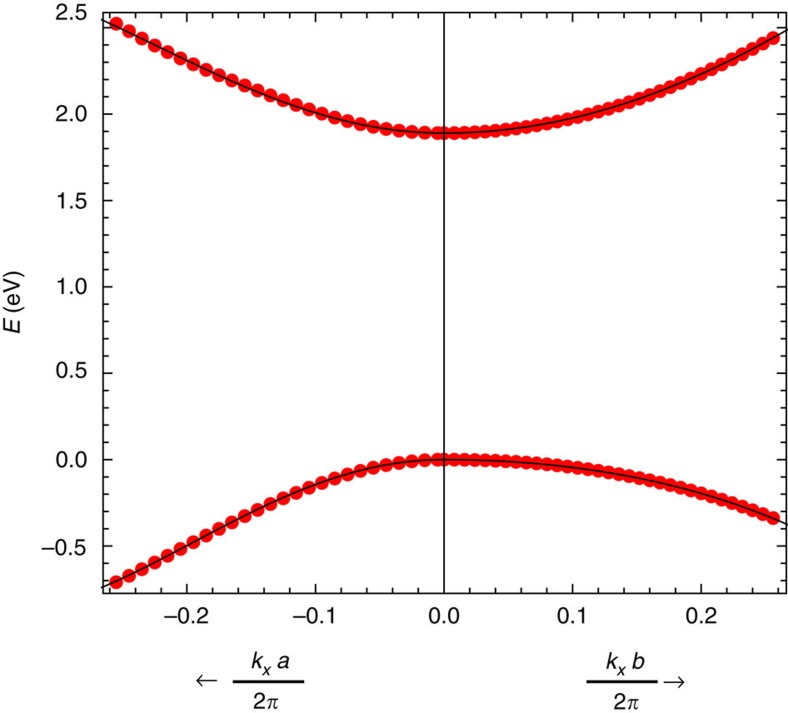
Tight-binding fit to *ab initio* for GeS. Dispersion of conduction and valence bands of GeS near Γ computed *ab initio* (red dots). A black line shows the tight-binding fit for comparison.

Owing to the lack of tight-binding models for monochalcogenide materials^46^,^47^, we have derived the tight-binding parameters by fitting the electronic structure of GeS *ab initio*. We used the PBE^72^ approximation to the exchange correlation functional, ultrasoft pseudopotentials^73^, Quantum-ESPRESSO^74^ and Wannier90 (ref. 75) computer packages. The cutoff for electron wavefunction is set to 40 Ry and cutoff for electron density to 200 Ry. Internal coordinates and in-plane lattice constants were fully relaxed. Vacuum region between repeating images of GeS monolayers is 17 Å. Wannier functions were constructed from a 12 × 12 regular k-mesh grid. The maximally localized Wannier functions were constructed in a standard way by projecting into hydrogenic *s*-like and *p*-like orbitals on both Ge and S atoms along with two *s*-like orbitals in the vacuum region that are needed to represent the vacuum states. The frozen window for the disentanglement procedure spans up to 6.2 eV above the Fermi level. The crystal structure of GeS is orthorombic with space group Pnma (No. 62) and lattice vectors 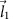
=(*l*_1_, 0) and 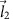
=(0, *l*_2_), with *l*_1_=4.53 Å and *l*_2_=3.63 Å and contains two Ge and two S atoms. The structure can be seen as two GeS zigzag chains separated by a height of *h*=2.32 Å. The *ab-initio* results for the conduction and valence bands near the Γ point are shown in Fig. 4 and have mostly *p*_*z*_ character.

This system can be effectively described with a two site tight-binding model. This can be done because the lattice structure has glide symmetries with mirror reflection *z*→−*z* and translations 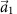
=(*a*_*x*_, *a*_*y*_) and 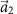
=(*a*_*x*_, −*a*_*y*_), with *a*_*x*_=*l*_1_/2 and *a*_*y*_=*l*_2_/2. When the out of plane positions of the atoms are not relevant for the problem of interest, one can define a smaller two site unit cell where the glides play the role of lattice vectors (as it is done in black phosphorus^69^). The Ge and S sites in this effective tight-binding model are located at (0, 0) and (*x*_0_, 0), with *x*_0_=0.62 Å. This is the tight-binding model employed in the main text. The parameters of this model are obtained from the *ab-initio* calculation as follows.

Since our aim is to model faithfully only the low-energy bands around the Gamma point, it will suffice to consider a single *p*_*z*_ orbital per site in the tight-binding model. The minimal model parameters are the on-site potential difference Δ between Ge and S *p*_*z*_ orbitals and the three nearest neighbours hoppings *t*_*i*_, with *i*=1, 2, 3, which are all between Ge and S atoms. In addition, to reproduce the small particle–hole asymmetry of the gap, we also consider two further neighbour hoppings 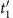 and 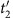, which connect Ge–Ge or S–S pairs (we assume the same values for both species to simplify).

The tight-binding Hamiltonian takes the form *H*=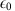
+Σ_*i*_*σ*_*i*_*f*_*i*_(**k**) with coefficients


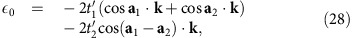



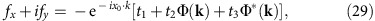



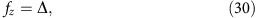


where, as defined in the text, 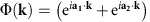. Our tight-binding fit is intended to reproduce faithfully the bands and wavefunctions close to the band edge, where the effective low-energy model applies. This model is given by


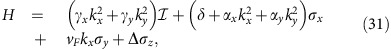


where a constant term is omitted as it can be absorbed in the chemical potential. The effective model parameters are related to the tight-binding parameters as


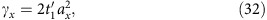



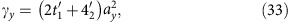



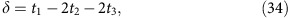



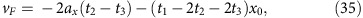



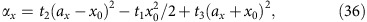



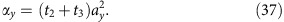


The key to obtain a reliable tight-binding parametrization is that, since the shift current depends sensitively on the actual wavefunctions, the tight-binding model should be fitted to wavefunction-dependent quantities in addition to the band energies. The simplest gauge invariant quantity that depends on wavefunction phases is the bracket of two covariant derivatives


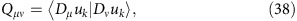


with *D*_*μ*_=∂_*μ*_−*iA*_*μ*_, with 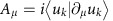 the Berry connection. The real and imaginary parts of this tensor are known as the Berry curvature and the quantum metric. A fit that reproduces this tensor correctly in addition to band energies ensures that the wavefunction structure around the Γ point is correctly accounted for, so that any other gauge invariant quantity computed in the effective model should be the same as that computed *ab initio*.

The Berry curvature Ω(*k*) is defined as


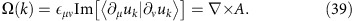


The Berry curvature around Γ for the tight-binding model is given by


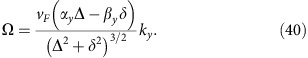


Since Ω vanishes at the origin, we take ∂_*y*_Ω as one extra input for the fit. The quantum metric is defined as


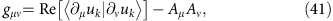


The only non-vanishing component of the quantum metric at *k*=0 is given by


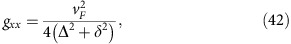


so we take *g*_*xx*_ as another extra input for the fit.

In summary, we take as *ab-initio* input parameters the gap, the four effective masses and the lowest order Berry curvature and quantum metric, ∂_*y*_Ω and *g*_*xx*_. The difference in effective masses for electron and hole bands, accounted for the term 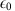, can be fitted independently with the hoppings 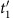 and 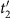. Since 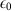 has no impact in the shift current response, the hoppings 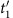 and 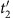 are not considered in the main text. The rest of the input is fitted with *t*_1_, *t*_2_ and *t*_3_, the on-site potential Δ and *x*_0_, and the results of the fit are shown in Table 1. While *x*_0_ is in fact known from the lattice structure of GeS to be 0.62 Å, obtaining it independently from the tight-binding fit, which gives a close value of 0.52 Å provides an additional check of the validity of the model.

### Data availability

The data that support the findings of this study are available from the corresponding author upon request.

## Additional information

**How to cite this article:** Cook, A. M. *et al*. Design principles for shift current photovoltaics. *Nat. Commun.* **8,** 14176 doi: 10.1038/ncomms14176 (2017).

**Publisher's note**: Springer Nature remains neutral with regard to jurisdictional claims in published maps and institutional affiliations.

## Figures and Tables

**Table 1:** *Ab initio* and tight-binding parameters for GeS.

*Ab-initio input parameters*						
*E*_g_	*m*_*x*,*v*_	*m*_*x,c*_	*m*_*y,v*_	*m*_*y,c*_	*∂*_*y*_ Ω	*g*_*xx*_
1.89 eV	−0.064 eV^−1^ Å^−2^	0.079 eV^−1^ Å^−2^	−0.340 eV^−1^ Å^−2^	0.171 eV^−1^ Å^−2^	3.565 Å^3^	2.529 Å^2^
						
*Tight-binding parameters*						
Δ	*t*_1_	*t*_2_	*t*_3_	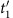	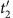	*x*_0_
0.41 eV	−2.33 eV	0.61 eV	0.13 eV	0.07 eV	−0.09 eV	0.52 Å

First row: input *ab-initio* parameters and the second row: tight-binding parameters obtained from the fitting.
